# Regulation of Catalyst Immediate Environment Enables Acidic Electrochemical Benzyl Alcohol Oxidation to Benzaldehyde

**DOI:** 10.1021/acscatal.4c00476

**Published:** 2024-03-29

**Authors:** G. Shiva Shanker, Arnab Ghatak, Shahar Binyamin, Rotem Balilty, Ran Shimoni, Itamar Liberman, Idan Hod

**Affiliations:** Department of Chemistry and Ilse Katz Institute for Nanoscale Science and Technology, Ben-Gurion University of the Negev, Beer-Sheva 8410501, Israel

**Keywords:** catalyst microenvironment, electrocatalysis, UiO-66, metal−organic framework (MOF), intermediate binding

## Abstract

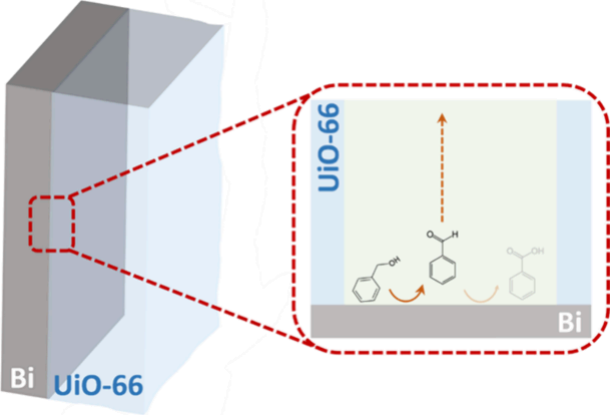

Electrocatalytic alcohol oxidation in acid offers a promising alternative to the kinetically sluggish water oxidation reaction toward low-energy H_2_ generation. However, electrocatalysts driving active and selective acidic alcohol electrochemical transformation are still scarce. In this work, we demonstrate efficient alcohol-to-aldehyde conversion achieved by reticular chemistry-based modification of the catalyst’s immediate environment. Specifically, coating a Bi-based electrocatalyst with a thin layer of metal–organic framework (MOF) substantially improves its performance toward benzyl alcohol electro-oxidation to benzaldehyde in a 0.1 M H_2_SO_4_ electrolyte. Detailed analysis reveals that the MOF adlayer influences catalysis by increasing the reactivity of surface hydroxides as well as weakening the catalyst-benzaldehyde binding strength. In turn, low-potential (0.65 V) cathodic H_2_ evolution was obtained through coupling it with anodic benzyl alcohol electro-oxidation. Consequently, the presented approach could be implemented in a wide range of electrocatalytic oxidation reactions for energy-conversion application.

## Introduction

Electrochemical alcohol oxidation is considered as an attractive replacement to the kinetically sluggish oxygen evolution reaction (OER) in water-splitting and fuel cell devices.^1−4^ As opposed to the large required overpotentials to drive water oxidation, electro-oxidation of alcohols generally necessitates lower applied potentials and thus, in principle, allows a more energetically efficient electrochemical production of H_2_. In particular, when working under acidic conditions, finding suitable OER catalysts is even more challenging as currently, only precious metal-oxide-based (Ir and Ru) materials can couple high activity and durability.^5−8^ In addition, there is a need to develop selective electrochemical alcohol transformation as it also offers the possibility to produce added-value chemicals.^9−13^

Particularly, benzyl alcohol (BnOH) electro-oxidation reaction to generate benzaldehyde (BnCHO) is of high interest due to its extensive use in pharmaceutical and agrochemical industries, fine chemical synthesis, and perfumery.^14−16^ Generally, the kinetics of electrochemical BnOH oxidation is faster in aqueous alkaline electrolytes compared to acidic and neutral mediums.^17^ However, the major product obtained in alkaline electrolytes is benzoic acid (BnCOOH) owing to the easier electro-oxidation nature of aldehydes over alcohols under these experimental conditions.^17^ On the other hand, in acidic solutions, it is found that the electro-oxidation kinetics of alcohols is sluggish in nature and requires the use of noble metal-based catalysts.^18,19^ Thus, the development of an earth-abundant catalyst for the selective electrosynthesis of aldehyde from its corresponding alcohol is still a major challenge. Recently, to improve the rate and selectivity of electrocatalytic reduction reactions (e.g., CO_2_ reduction and H_2_ evolution), an alternative approach has recently been explored, utilizing reticular chemistry to modulate the catalyst’s microenvironment. In this conceptual design, the catalyst surface is modified by a thin overlayer of a functional porous material such as covalent–organic frameworks and metal–organic frameworks (MOFs).^20−23^ These layered coatings affect catalysis occurring at the underlaying electrocatalyst through (i) altering the flux of catalytic substrates/ions proximal to the active sites and (ii) tuning the reactive intermediate binding during electrocatalysis. Nevertheless, to the best of our knowledge, no attempt has been made to use the porous overlayer coating approach to tune oxidative electrochemical transformation of organic substrates.

In this report, as a model system, we chose to study BnOH electro-oxidation using a bismuth (Bi) electrocatalyst in an acidic medium. Previously, Bi was used as an electrocatalyst for the oxidation of organic molecules.^24,25^ Moreover, Bi/bihydroxide was used as a promotor alongside additional metal (M = Pt abd Pd) (MBi_*x*_) oxidation of different alcohols including BnOH, albeit to moderate selectivities.^26−32^ However, selective oxidation of BnOH to the desired product remains challenging. Hence, to test our approach, we have coated a Bi-based electrocatalyst with a thin layer of Zr_6_-oxo-based MOF, UiO-66.^33^ Thereafter, we studied the manner in which the MOF coating affects the system’s activity and selectivity toward electrochemical oxidation of BnOH to BnCHO. It was found that the electrochemical BnOH oxidation performance of Bi was profoundly enhanced by the MOF overlayer, exhibiting improvement of selectivity (from 60 to 98%) and activity (1.73 times higher currents) at 0.45 V vs RHE. Detailed mechanistic analysis, done using operando FTIR spectroscopy and isothermal titration calorimetry (ITC), reveals that the UiO-66 layer (i) provides electrophilic hydroxide species (located at the Zr_6_-oxo MOF node as well as on the catalyst’s surface) that facilitates BnOH oxidation to BnCHO and (ii) suppresses further oxidation of BnCHO by weakening its binding to the catalyst’s surface. Additionally, we demonstrate that H_2_ could be generated at a low applied potential of 0.65 V by coupling the anodic electrochemical BnOH oxidation reaction to a cathodic hydrogen evolution reaction in a two-electrode configuration.

## Results and Discussion

The UiO-66 thin films were coated on Bi foil via the preparation of an MOF gel precursor following a previously published UiO-66 gel preparation procedure.^34^ Briefly, the synthesis procedure was as follows: Zirconium oxychloride hexahydrate and 1,4-benzene dicarboxylic acid were mixed in *N*,*N*-dimethylformamide (DMF) along with acetic acid and HCl. The mixture was sonicated for 5 min to make a clear solution and then kept at 100 °C in a hot oven for 2 h. Next, the DMF was additionally added to the reaction mixture, and the mixture was homogenized and kept at 120 ^o^ C in a hot oven for 24 h. Subsequently, the obtained UiO-66 gel was washed with ethanol (see the Experimental Section in the Supporting Information (SI) for synthesis details). The as-synthesized UiO-66 gel was used as a precursor to grow a thin layer of Bi electrocatalyst. Such a thin layer was termed a membrane and is noted as Bi-UiO-66. Then, such a Bi-UiO-66 catalyst was characterized using structural, chemical, and microscopic techniques and further used in all electrochemical measurements.

**Figure 1:**
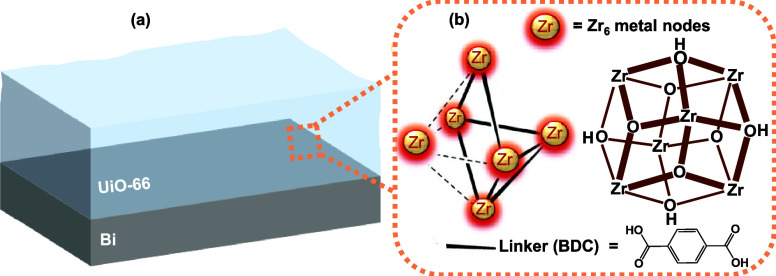
(a) Schematic illustration of a UiO-66 membrane assembled on a Bi electrocatalyst. (b) Representation of the UiO-66 structure.

**Figure 2:**
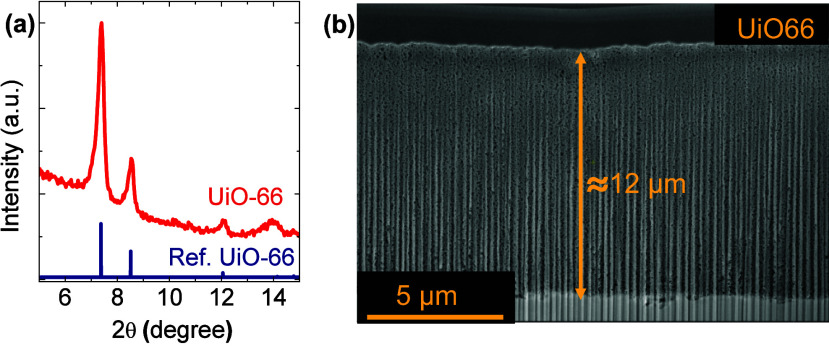
(a) PXRD pattern of the UiO-66 membrane in comparison with reference data of UiO-66. (b) SEM-FIB cross section image of the UiO-66 membrane on the Bi electrocatalyst.

After the synthesis, as depicted in the pictorial image (Figure 1), the UiO-66 membrane is grown on a flat Bi foil electrocatalyst by drop casting. The structural information on the UiO-66 membrane on the Bi electrocatalyst is obtained using powder X-ray diffraction (PXRD) pattern. All the diffraction peaks of the UiO-66 membrane in the PXRD pattern are well matched with the reference data of UiO-66 (Figure 2a), which conveys that the UiO-66 thin film has been successfully grown on the Bi electrocatalyst. As shown in Figure 2b, the thickness of the UiO-66 membrane on the Bi electrocatalyst is estimated to be ∼12 μm using cross-sectional scanning electron microscopy–focused ion beam (SEM-FIB). In addition, SEM images showed a uniform coating of UiO-66 over the Bi surface, as shown in Figure S1a–c. Further, to analyze the elemental composition of the Bi-UiO-66 membrane, as shown in Figure S2, and to confirm that the UiO-66 membrane covers the underlying Bi surface, the X-ray photoelectron spectroscopy (XPS) survey spectrum of Bi-UiO-66 was measured, showing the existence of UiO-66 elements: Zr, C, and O (red). Upon ion-beam etching of the MOF, Bi was also detected (navy blue). Moreover, the nature of the Bi catalyst surface was unchanged after the formation of the UiO-66 membrane, as confirmed with XPS analysis of Bi 4f peaks (Figure S3).

To quantify the amount of 1,4-benzene dicarboxylic acid (BDC) linker per Zr_6_-oxo node in the UiO-66 membrane, we have combined both proton nuclear magnetic resonance (^1^H NMR) and inductively coupled plasma optical emission spectrometry (ICP-OES). Specifically, the amount of BDC linkers was determined using ^1^H NMR of digested UiO-66 in 1 M NaOH D_2_O solution (Figures S4 and S5). Similarly, the amount of Zr in UiO-66 was quantified using ICP-OES by digesting a known amount of UiO-66 in concentrated HNO_3_ (see the Experimental Section in the SI for details). The obtained BDC/Zr_6_ ratio in our UiO-66 is ∼8, meaning that our MOF-node structure contains four missing BDC linkers compared to an ideal, 12-coordinated UiO-66 structure and thus exposes a large concentration of terminal hydroxyl groups.^35^

Upon characterization of the MOF membrane, we were set to investigate the effect of UiO-66 coating on Bi’s electrocatalytic performance toward oxidation of BnOH. All the electrochemical experiments were performed in a two compartment H-cell with a conventional three-electrode design in which the reference (Ag/AgCl (3 M KCl)) and working electrode (Bi foil or Bi-UiO-66) are separated from the counter electrode (Pt foil) by a Nafion-117 ion-exchange membrane. All electrochemical experiments were conducted in a 0.1 M H_2_SO_4_ aqueous electrolyte solution at a scan rate of 50 mV/s and under an Ar environment.

**Figure 3:**
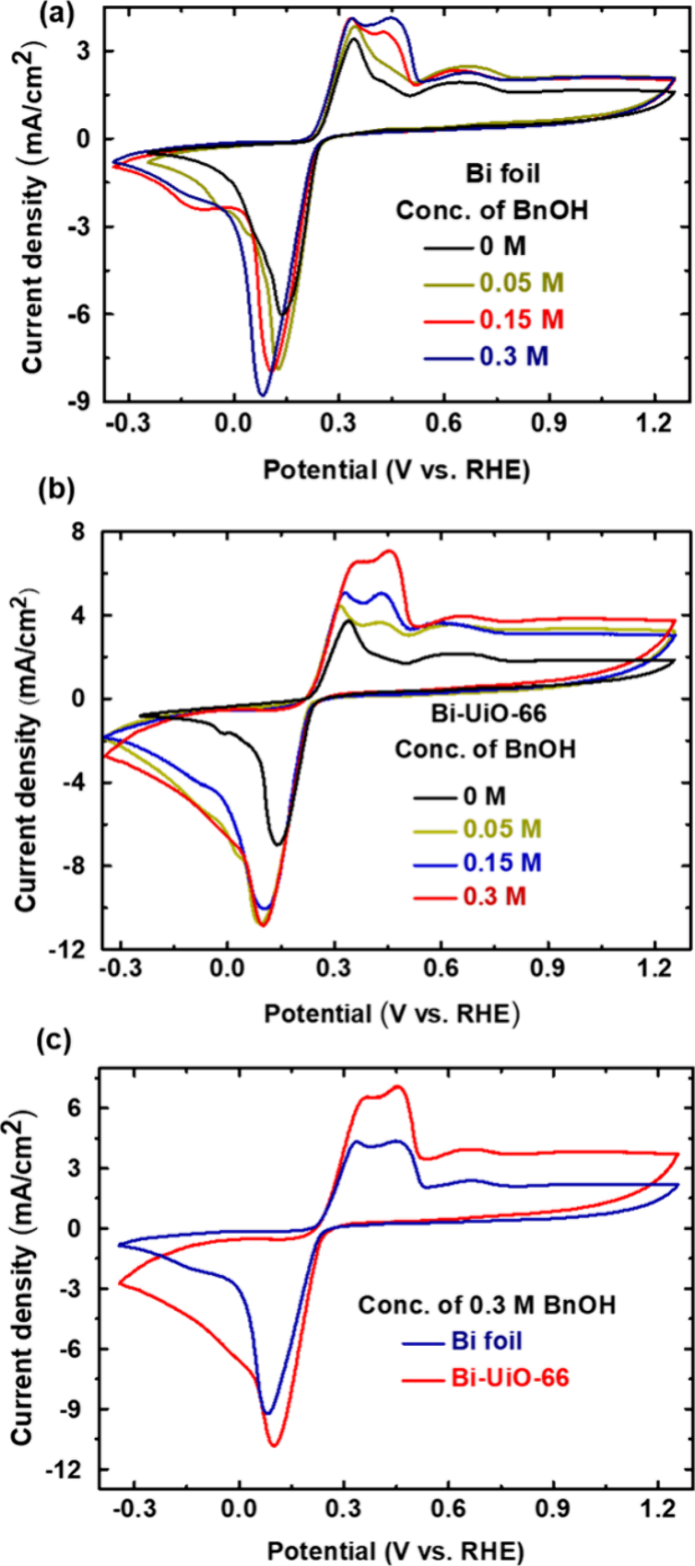
Cyclic voltammograms of (a) Bi and (b) Bi-UiO-66 electrocatalysts measured with different concentrations of benzyl alcohol. (c) Comparison of CVs of Bi and Bi-UiO-66 in 0.3 M benzyl alcohol-containing electrolyte.

First, we investigate the redox properties of Bi foil and UiO-66 membrane-coated Bi (Bi-UiO-66) foil using cyclic voltammetry (CV). As shown in Figure S6, scanning the potential in the anodic direction without added BnOH in the solution, Bi and Bi-UiO-66 exhibit two oxidation peaks located at 0.34 (peak I) and 0.45 V (peak II) vs RHE, corresponding to the formation of Bi-OOH/Bi(OH)_3_, and an additional broad peak centered at 0.64 V vs RHE (peak III) was attributed to further oxidation to Bi_2_O_3_, consistent with prior reports.^25^Figure 3a shows CVs of Bi foil measured in the presence of different BnOH concentrations (0–0.3M). Interestingly, for peak I, oxidation currents only slightly increase with the addition of BnOH as compared to the absence of BnOH. In other words, at these potential ranges, Bi foil exhibits limited kinetics for BnOH electro-oxidation. However, at potentials corresponding to peak II, the current increases gradually as a function of BnOH concentration as a result of its electro-oxidation by Bi-OOH (which is formed at the same potential region). The oxidation current decays at potentials above ∼0.5 V vs RHE due to the blocking of catalytic sites by formation of a fully oxidized BiO_*x*_.^25^ CV experiments were also conducted on Bi-UiO-66 (Figure 3b). As opposed to Bi foil, in the presence of a UiO-66 adlayer, the BnOH oxidation current of both peaks (I and II) is enhanced. Furthermore, as seen in Figure 3c, when comparing the BnOH oxidation activity of both samples, Bi-UiO-66 exhibits significantly enhanced oxidation kinetics (a current increase of 73% at 0.45 V vs RHE).

**Figure 4:**
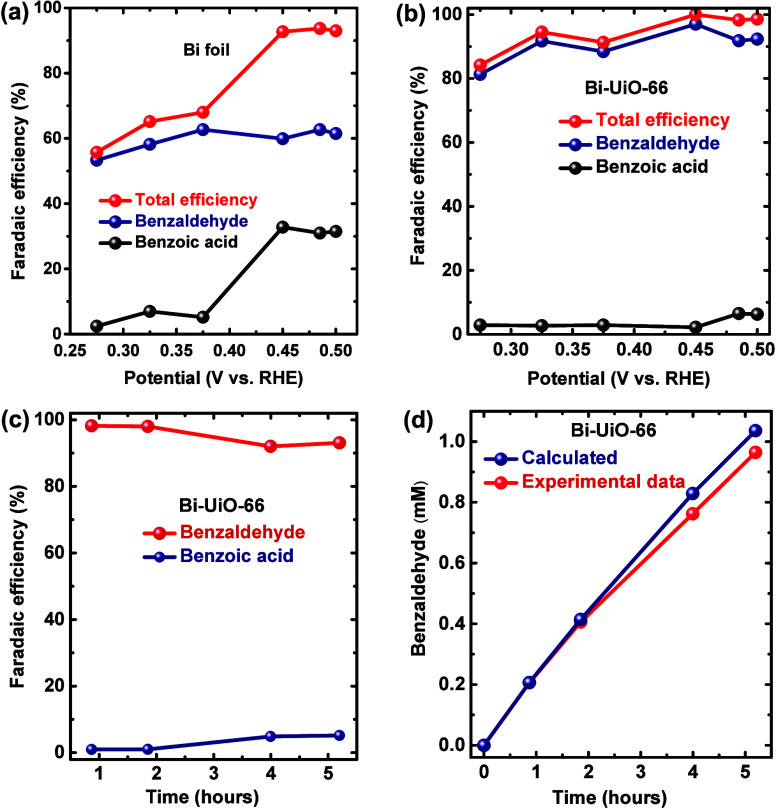
Variation in BnOH oxidation FE of (a) Bi and (b) Bi-UiO-66 electrocatalyst for BnCHO and BnCOOH at different applied potentials. (c) FE of Bi-UiO-66 during 5 h of electrolysis and (d) comparison of experimentally produced BnCHO with theoretical values.

Next, to examine the electrocatalytic BnOH oxidation selectivity of Bi and Bi-UiO-66, we have performed a set of bulk electrolysis experiments at potentials range of 0.275–0.5 V vs RHE in a 0.1 M H_2_SO_4_ solution containing 0.3 M BnOH (see chronoamperometric curves in Figures S7 and S8). Electro-oxidation products were determined using ^1^H NMR analysis of the reaction’s electrolyte samples via comparison with calibration data of BnCHO and BnCOOH (Figures S9–12). As shown in Figure 4a, for Bi foil, BnCHO and BnCOOH are the major products determined for BnOH oxidative electrolysis. The obtained faradaic efficiency (FE) for the BnCHO formation was ∼60% throughout the entire potential range. Notably, at potentials below 0.375 V vs RHE (i.e., peak I), only small amounts of BnCOOH are formed, with an obtained FE of less than 10%.

However, at higher potentials (peak II), BnCOOH FE increased to ∼30%. On the other hand, at all potentials, Bi-UiO-66 exhibits high selectivity toward BnCHO (FE of 81–98%), with only trace amounts of BnCOOH (Figure 4b). In other words, one can clearly see that the application of the UiO-66 membrane affects electrocatalytic BnOH oxidation by (a) significantly enhancing the selectivity toward BnCHO formation and (b) substantially increasing the total FE for BnOH oxidation at potentials corresponding to peak I (consistent with UiO-66’s activation of peak I toward BnOH oxidation observed in the CVs of Figure 3a,b).

We then were interested in finding the optimum UiO-66 thickness on Bi foil and hence have prepared three different Bi-UiO-66 samples with varying thicknesses (5.8, 12, and 19 μm) by changing the loading amount of the UiO-66 gel precursor (Figure S13). CV and bulk electrolysis experiments (conducted at 0.45 V vs RHE) of these samples suggested that the 12 μm-thick UiO-66 adlayer exhibits the highest electrocatalytic BnOH oxidation activity and selectivity (Figures S14 and S15). These results suggest that the mass transport rate of the catalytic substrate (BnOH) is attenuated for the case of the highest thickness of UiO-66, thus resulting in a decrease in alcohol oxidation current. Hence, the 12 μm-thick UiO-66 coating on Bi foil is considered the optimum thickness for this reaction, and thus it was further used throughout the manuscript and termed as Bi-UiO-66.

After the excellent catalytic activity and selectivity of Bi-UiO-66 for the alcohol oxidation reaction, it is essential to identify the active site on the catalyst surface. We have analyzed the surface composition of Bi-UiO-66 using XPS after electrolysis at 0.45 V vs RHE (passing a 1.5 C charge). After electrolysis, first, the electrode was washed thoroughly with double-distilled water. Then, the electrode was dried in a vacuum oven and stored in an Ar environment to avoid any surface oxidation of the catalyst until subjected to the XPS measurement. Figure S16a shows the Bi 4f spectrum, with 4f_7/2_ peaks at binding energies of 157.5, 158.6, 159.7, and 160.6 eV assigned as metallic Bi, Bi(OH)_3_, Bi-OOH, and Bi_2_O_3_, respectively (as well as their corresponding satellite 4f_5/2_ peaks at 162.8, 163.9, 165.05, and 165.9 eV), in agreement with prior reports.^36^ In other words, upon electrochemical operation, a noticeable decrease in the amount of metallic Bi was detected compared to that before electrolysis (Figure S3). In turn, the metallic Bi was converted mainly to Bi-OOH (the largest peak located at 158.6 eV is attributed to Bi-OOH). Additionally, the O 1s spectrum (Figure S16b) shows peaks at binding energies of 530.8 and 532.3, which can be assigned to Bi–O and Bi–OH, respectively, suggesting that Bi^3+^ is mainly in the form of Bi-OOH similar to the prior literature.^29^ Thus, it indicates that Bi-OOH constitutes the catalytically active species that is involved in the alcohol oxidation reaction.

Subsequently, to check the stability of Bi-UiO-66, we employed a chronoamperometric experiment (Figure S17) at its optimal catalytic activity (0.45 V vs RHE) for 5 h. As seen in Figure 4c, the FE of BnCHO is retained above 93% even after 5 h of bulk electrolysis. Hence, the amount of experimentally produced BnCHO nearly equals the theoretically calculated amount throughout 5 h of electrolysis (Figure 4d). Moreover, PXRD and SEM analyses of Bi-UiO-66 after 5 h of bulk electrolysis show the retainment of the UiO-66 structure and surface morphology (Figures S18 and 19). The SEM-FIB cross-sectional image (Figure S20) shows that the UiO-66 film thickness (11.5 μm) is also maintained. Moreover, diffuse reflectance infrared Fourier transform spectroscopy characterization (Figure S21) reveals the retainment of the typical stretching vibrations of the MOF’s terminal −OH groups at the Zr_6_-oxo node (3648 cm^–1^) and surface-bound −OH groups on Bi (3612 cm^–1^), indicating the structural integrity of Bi-UiO-66 after 5 h of electrolysis.

**Figure 5:**
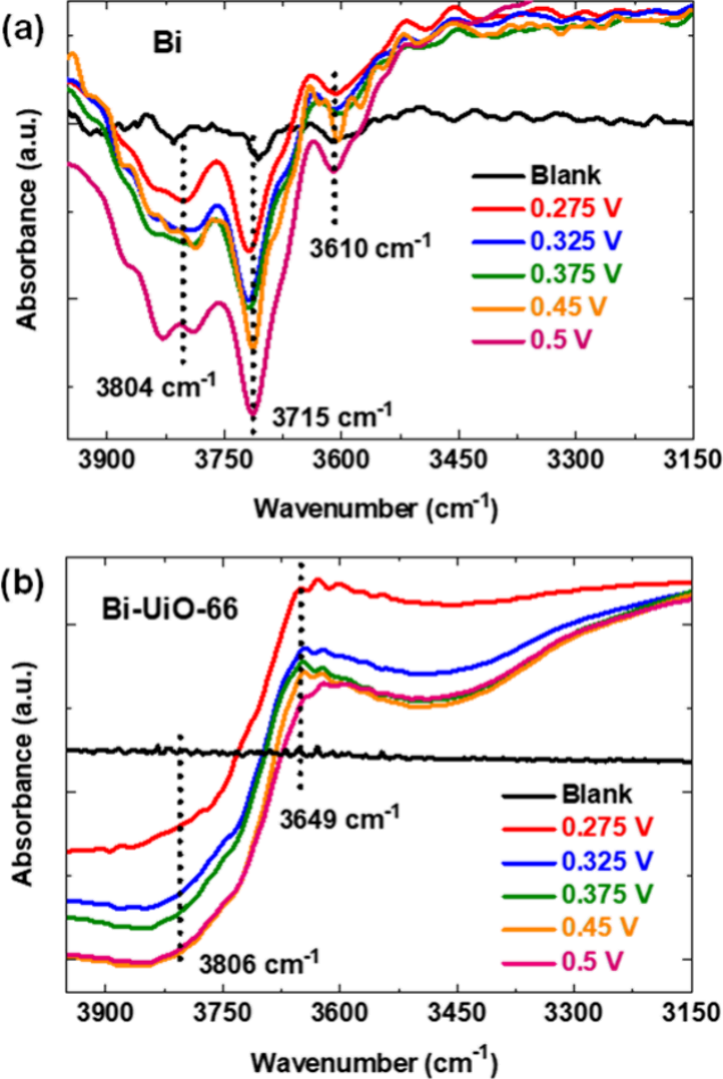
Operando electrochemical ATR-IRRAS spectra of (a) Bi and (b) Bi-UiO-66 under electrochemical BnOH oxidation conditions.

In order to understand the role of UiO-66 in the improved electro-oxidation kinetics of BnOH, spectro-electrochemical analysis has been performed over Bi and Bi-UiO-66 electrodes under the same electrocatalytic conditions using operando infrared reflection absorption spectroscopy (ATR-IRRAS) in Otto configuration.^37−39^ For the Bi foil electrode, with the application of anodic potentials from 0.275 to 0.5 V (i.e., where BnOH oxidation takes place), a gradual growth in the three negative bands is observed at 3610, 3715, and 3804 cm^–1^ (Figure 5a). These bands correspond to perturbation of surface, Bi-bound hydroxyl groups,^40,41^ thus suggesting that the surface-bound hydroxyl groups are consumed in the course of the catalytic oxidation reaction. Importantly, for these Bi-bound −OH bands, a higher IR frequency implies for stronger hydroxyl basicity, i.e., the 3804 cm^–1^ −OH species possess the most basic nature.^42^ This allows us to understand the role of surface-bound hydroxyl groups in the electro-oxidation of BnOH, which could facilitate the reaction through the abstraction of the alcohol’s α-proton by a more basic Bi-based hydroxyl group. Figure 5b presents the IR spectra of Bi-UiO-66 under the same experimental conditions. Interestingly, the presence of the MOF membrane alters the electrocatalytic kinetics via two distinctive manners: (a) Under positive applied potentials, a gradually growing negative peak located at 3806 cm^–1^ is detected, attributed to the highly reactive, basic Bi-bound −OH groups. In general, increased surface coverage by an IR probe moiety (e.g., −OH) can lead to interaction among them, causing a shift in the peak position. The present case is not an exception to that as well. After the incorporation of UiO-66 MOF, there is indeed a possibility of interaction between the MOF’s Zr_6_-oxo node and −OH groups on the Bi surface, i.e., surface coverage effect, which may cause a shift in the peak position. Hence, it is reasonable to assume that the occurrence of the broad band at 3806 cm^–1^ for Bi-UiO-66 is a result of both effects: (i) increase in −OH basicity and (ii) surface coverage. In other words, we can even further add that the increase in basicity of −OH groups is a consequence of the rising interaction of −OH groups with the MOF due to surface coverage effect. Having said that, we also cannot completely rule out the effect of changes to catalyst surface structure on the resulting IR data. Nonetheless, the more acidic Bi-based −OH species (bands at 3610 cm^–1^, 3715 cm^–1^) could not be detected. In other words, the MOF coating increases the population of highly reactive, electrophilic surface hydroxyls on the expense of less active, acidic ones. (b) The UiO-66 MOF exposes Zr_6_-oxo node-based terminal −OH groups at close proximity of the catalyst surface observed by the 3649 cm^–1^ IR band.^35^ Again, under applied positive potential, the 3649 cm^–1^ band also decreases in intensity, thus signaling the consumption of MOF-node’s hydroxides during the catalytic reaction. Therefore, it indicates that in the case of Bi-UiO-66, both UiO-66 and Bi-bound −OH groups synergistically aid catalysis, which further improves BnOH’s electro-oxidation kinetics.

**Figure 6:**
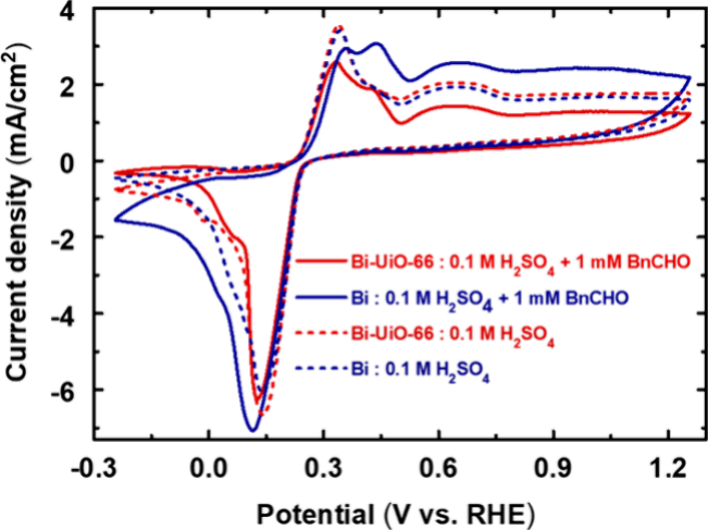
Comparison of CV data of Bi and Bi-UiO-66 electrocatalyst in 1 mM BnCHO containing 0.1 M H_2_SO_4_ aqueous solution.

Then, upon disclosing the manner in which the MOF membrane enhanced the catalysis rate, we were interested in understanding the factors controlling BnCHO selectivity improvement of Bi-UiO-66 compared to Bi foil. To do so, we intentionally chose BnCHO as a catalytic substrate and performed the following experiments. First, for both Bi foil and Bi-UiO-66, we have conducted CVs in the electrolyte solution with and without 1 mM BnCHO. As seen in Figure 6, in the absence of BnCHO, the electrochemical response of Bi foil and Bi-UiO-66 is similar (dotted curves). Yet, when BnCHO is added to the electrolyte, Bi foil shows increased catalytic oxidation activity at potentials attributed to peak II, centered at 0.43 V vs RHE (blue line), whereas such a catalytic rise in oxidation peak II has not emerged for Bi-UiO-66 (red line). As such, it indicates that unlike Bi foil, the UiO-66 membrane suppresses electro-oxidation of BnCHO to BnCOOH. We note that for both samples, we observe the diminishing of oxidation peak I in the presence of BnCHO, in agreement with their lack of ability to oxidize BnOH to BnCOOH at these potentials (Figure 4a,b). Consequently, we postulated that the observed catalytic selectivity might be controlled by attenuated reaction intermediate (BnCHO) adsorption strength over the catalyst’s surface.^43^

**Figure 7:**
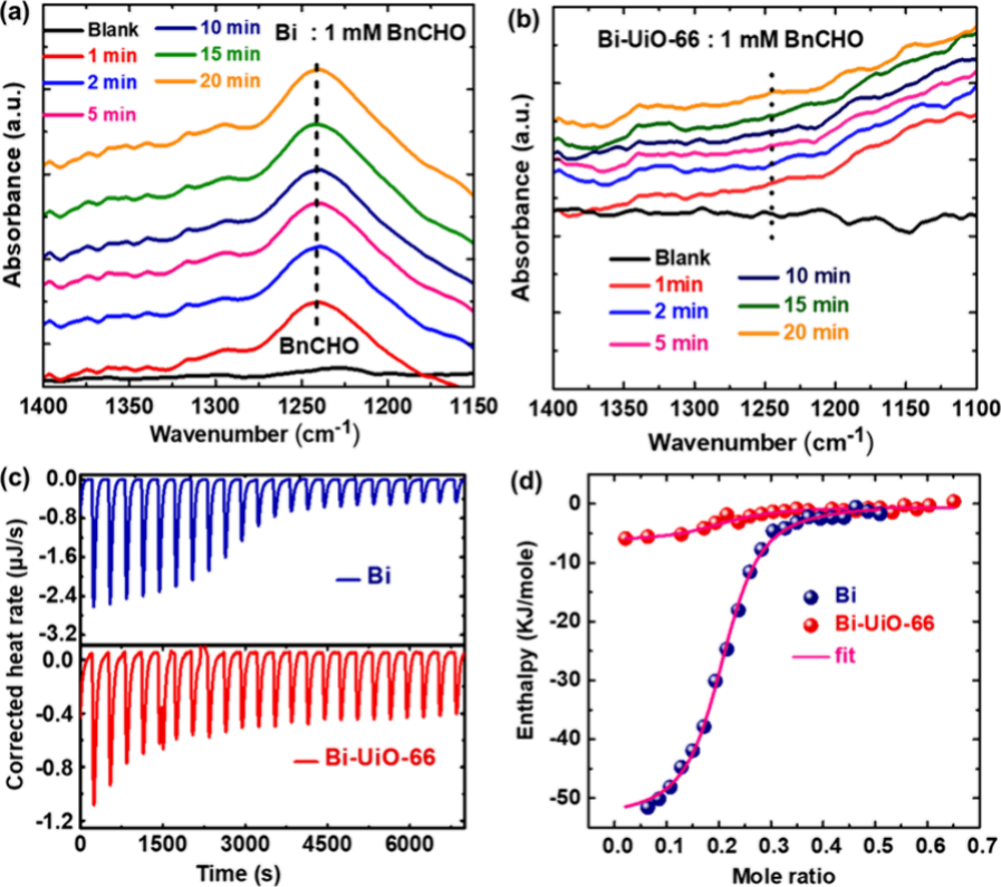
Time-dependent ATR-IRRAS measurements of (a) Bi and (b) Bi-UiO-66 in an electrolyte containing 1 mM BnCHO in 0.1 M H_2_SO_4_. ITC of (c) Bi (blue) and Bi-UiO-66 (red) catalyst (0.05 mM). Data from automatic injections of 5 μL portions of BnCHO (0.5 mM) into a catalyst-containing cell. (d) Plot of the total heat released as a function of BnCHO concentration for the titration (the pink line denotes the best fits).

Hence, to monitor the adsorption strength of BnCHO over Bi and Bi-UiO-66, we employed ATR-IRRAS spectroscopy. Measurements were conducted in 0.1 M H_2_SO_4_ solutions containing 1 mM BnCHO by recording IR spectra of the catalyst’s surface over time. As seen in Figure 7a, for bare Bi, an IR band appears at 1240 cm^–1^, which is attributed to the formation of BnCHO adsorbed on the surface. This specific band appears due to the mixed vibrational mode consisting of ring COH stretching (ν(ph-COH)), aldehydic CH bending (δ(C–H)_ald_), CH bending of the ring (δ(C–H)_ring_), and C=C stretching of the ring (ν(C–C)_ring_), commensurate with a surface-bound BnCHO.^44−47^ Similarly, for bare Bi, a band appears at 1718 cm^–1^, which is also attributed to the −C=O stretch of aldehyde according to previous literature reports^40,48^ (Figure S22a). On the contrary, for Bi-UiO-66, such BnCHO adsorption IR features do not appear under the same experimental conditions (Figure 7b and Figure S22b), thus hinting at the fact that the MOF membrane weakens the surface binding affinity of BnCHO.

As a result, to gain further insights for the thermodynamics of BnCHO’s interaction with the catalyst, we have also conducted an ITC analysis.^49^ The resultant thermograms for titration of BnCHO solution into a suspension of the catalysts (Bi and Bi-UiO-66) exhibit negative signal peaks, indicative of exothermic interaction of the titrant (BnCHO solution) with the catalyst (Figure 7c). The obtained binding isotherms (Figure 7d) exhibit a sigmoidal curvature with binding stoichiometry corresponding to the chemical interaction between BnCHO and catalysts. By fitting the curves, one can extract the thermodynamic interaction parameters, as plotted in Table S1. Indeed, BnCHO chemisorption to bare Bi is substantially stronger compared to that of Bi-UiO-66, as evident by its seemingly larger BnCHO binding constant on Bi (*K*_a_ = 1.379 × 10^6^ M^–1^) compared to that of Bi-UiO-66 (*K*_a_ = 0.598 × 10^6^ M^–1^). Additionally, binding enthalpy values suggest that BnCHO interacts more favorably with bare Bi (Δ*H* = −53.4 kJ/mol) compared to Bi-UiO-66(Δ*H* = −5.89 kJ/mol).^49,50^ Accordingly, these obtained results point to the fact that the UiO-66 membrane improves the catalytic selectivity by weakening the binding of BnCHO to the Bi’s surface and thus in turn does not allow its further oxidation to BnCOOH.

**Figure 8:**
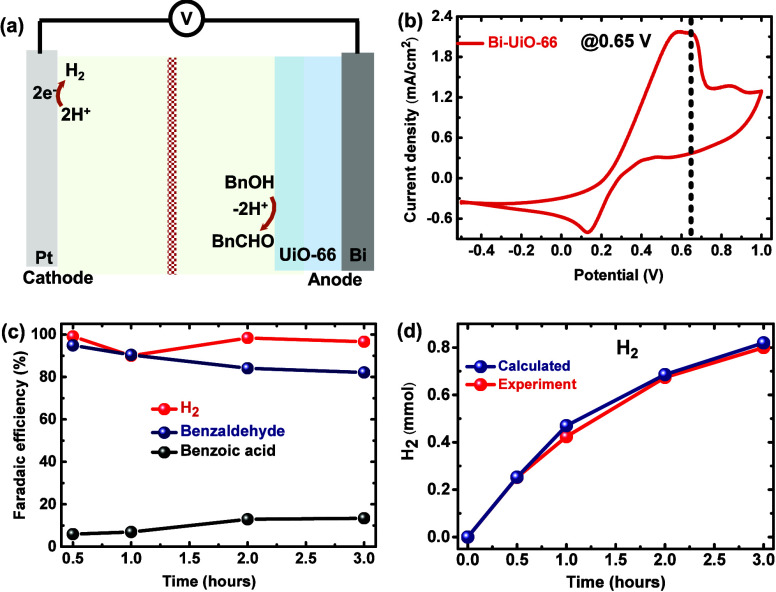
(a) Illustration of a two-electrode setup coupling BnOH oxidation with H_2_ evolution. (b) CV data of Bi-UiO-66 in a two-electrode setup. (c) Variation in FE of Bi-UiO-66 toward BnCHO, BnCOOH, and H_2_ generation at 0.65 V during 3 h of electrolysis. (d) Comparison of experimentally produced H_2_ with theoretical values.

By now, we have unveiled the excellent catalytic activity and selectivity of Bi-UiO-66 for oxidation of BnOH to BnCHO in acid. Subsequently, we then turned to test the notion of coupling the anodic electro-oxidation reaction with cathodic H_2_ evolution in order to replace the energy-demanding OER. To this end, we used a two-electrode electrochemical setup with an anodic compartment comprising the Bi-UiO-66 catalyst in a 0.1 M H_2_SO_4_ electrolyte containing 0.3 M BnOH and a cathodic compartment comprising Pt foil as the H_2_-evolving cathode in a 0.1 M H_2_SO_4_ electrolyte. Both compartments were separated by a Nafion-117 ion-exchange membrane (see Figure 8a).

Anodic CV data conducted with Bi-UiO-66 for BzOH oxidation are shown in Figure 8b. The catalytic oxidation peaks (I and II) are positively shifted due to uncompensated IR drop accruing at the cathodic half-cell. To quantify the amount of generated H_2_ at the cathode, we conducted a 3 h chronoamperometric bulk electrolysis measurement at 0.65 V (i.e., potential corresponding to peak II) (Figure S23). The produced H_2_ gas was quantified using gas chromatography (Figure S24). As shown in Figure 8c,d, the FE of H_2_ evolution is practically 100% (matching the theoretical values of 0.8 mmol produced H_2_), while the BnCHO FE remains above 80% after 3 h of electrolysis. Thus, Bi-UiO-66 allows low-potential, stable H_2_ generation coupled to selective BnOH oxidation to BnCHO. To check the stability of the catalyst in long-term electrolysis, we have also conducted a chronoamperometric experiment under the same experimental conditions, albeit the addition of BnOH into solution at different time intervals during electrolysis (Figure S25). As electrolysis progresses, one can observe a decrease in current density. However, the decreased current density is restored to its initial value upon injection of fresh BnOH to the electrolyte. This suggests that the recorded decrease in current density with time in long-term electrolysis is mainly due to the consumption of reactants and is not a result of catalyst deactivation.

## Conclusions

In this report, we have demonstrated that adjustment of the catalyst microenvironment facilitates efficient and selective electrocatalytic oxidation of BnOH to BnCHO under acidic conditions. Specifically, a non-electro-active MOF membrane (UiO-66) was coated over a Bi solid electrocatalyst. In this design, the UiO-66 membrane improved Bi’s electrocatalytic activity (currents enhanced by 73%) and selectivity (BnCHO FE reaching up to 98%) at 0.45 V vs RHE. Detailed electrochemical, spectroscopic, and calorimetric characterizations show that the MOF adlayer affects electro-oxidation through (i) accelerating the oxidation reaction by promoting the participation of electrophilic −OH moieties (positioned both at the Zr_6_-oxo MOF node and on the catalyst’s surface) and (ii) suppressing surface adsorption of the BnCHO intermediate, hence avoiding its further oxidation to BnCOOH. Notably, we were able to couple the anodic organic transformation reaction to a cathodic H_2_ evolution at a low potential of 0.65 V vs RHE. Consequently, this notion could in principle be utilized in other energy-related oxidative electrochemical systems.

## Supporting Information Available

Chemicals, experimental procedures, material synthesis and characterization, physical characterization methods, and details of electrochemical experiments (DOCX)
