# Supplementary material for: Regulation of Catalyst Immediate Environment Enables Acidic Electrochemical Benzyl Alcohol Oxidation to Benzaldehyde
Source: ACS Catal. 2024 Mar 29;14(8):5654–61. doi: 10.1021/acscatal.4c00476 (PMC11036388; doi:10.1021/acscatal.4c00476)
Supplement: Supplementary file 1 — cs4c00476_si_001.docx [file cs4c00476_si_001.docx]

**Supporting Information**

**Regulation of Catalyst Immediate Environment Enables Acidic Electrochemical Benzyl Alcohol Oxidation to Benzaldehyde**

G. Shiva Shanker, Arnab Ghatak, Shahar Binyamin, Rotem Balilty, Ran Shimoni, Itamar Liberman, and Idan Hod*

Department of Chemistry and Ilse Katz Institute for Nanoscale Science and Technology,
Ben-Gurion University of the Negev,
Beer-Sheva, 8410501, Israel.
E-mail: [hodi@bgu.ac.il](mailto:hodi@bgu.ac.il)

TABLE OF CONTENTS

| Sections | Page number |
| --- | --- |
| Table of Contents | S2 |
| Experimental Procedures | S2-S5 |
| Physical Characterization Methods | S5 |
| Results and Discussion | S6-S15 |

EXPERIMENTAL PROCEDURES

The Chemicals

N, N-dimethylformamide (DMF, C_3_H_7_NO), and acetic acid (AA, CH_4_CO_2_, ≥ 98%) were purchased from Bio-Lab. Nafion solution perfluorinated resin solution was purchased from Sigma-Aldrich. D_2_O solution (> 99.9 %) was purchased from Tzamal D-chem laboratories. Terephthalic acid (C_8_H_6_O_4_ ≥ 98%) and Zirconyl dichloride octahydrate (ZrOCl_2_·8H_2_O ≥ 99.5%) were purchased from Sigma-Aldrich. Ethanol (C2H_6_O,99.7%) was purchased from J.T Baker. Bi-foil (≥ 99%) was purchased from Holland Moran. Ar gas (≥ 98%) was purchased from Maxima.

Cleaning of Bi foil

Firstly, the Bi foils were cut into 2.5 cm x 1 cm pieces. These Bi foils were polished using alumina powder (3 µm) on a polishing cloth. Then, Bi foils were cleaned by sonicating in a soap solution, milli-q (mq) water, and ethanol, respectively, and dried in a vacuum oven to remove surface adsorbed species. Subsequently, the Bi electrodes were stored in an Ar environment. Before subjecting an experiment, Bi foils were washed with mq. water and dried in Ar flow. These Bi-foils were covered by insulating tape except for one top surface where the electrochemical reaction was performed. Thus, the uncovered surface of Bi foil was used to coat the UiO-66 membrane. Note that an insulating tape was electrochemically inactive in the potential window of our interest.

**Synthesis of UiO-66 gel**

UiO-66 gel was synthesized by following the previous report.[^1^](#_ENREF_1) In detail, ZrOCl_2_ (644 mg), BDC (482 mg), and acetic acid (400 μL) were taken in 12 mL DMF containing 100 mL reagent bottle with a Teflan screw cap. This solution mixture was subjected to sonication to form a clear solution. Then, the reagent bottle was kept in a programmable oven at 100 ^o^C for 120 min. The reaction mixture was naturally cooled to room temperature, forming a white color gel. Afterward, 12.5 mL DMF was added to the reaction mixture and homogenized by shaking with a vertex shaker. Then, the glass bottle was tightly screwed and kept it in the programmable oven at 120 ^o^C for 24 hours and cooled naturally to room temperature. Likewise, 25 mL of DMF was added to the reaction mixture, homogenized the solution using a vertex shaker, and kept in a programmable oven for 24 hours. After cooling the reaction mixture, we washed it with ethanol in a controlled manner to remove excess DMF from the reaction mixture as follows. 50 mL of ethanol was added to the reaction mixture, homogenized the solution, kept in the programmable oven at 60 ^o^C for 24 hours, and cooled to room temperature. The gel was settled in the bottom of a glass bottle, 50 mL solution was discarded from the top, 50 mL fresh ethanol was added to the reaction mixture, and this step was repeated thrice. This was considered as synthesized UiO-66 gel. To grow the UiO-66 membrane on Bi foil, we diluted the as synthesized UiO-66 gel using ethanol used as a precursor.

The 100 µL diluted UiO-66 gel precursor was drop cast on Bi foil in a single step, and the time required to form the UiO-66 membrane was 2 to 3 hours at the ambient conditions. UiO-66 coated Bi was named Bi-UiO-66 throughout the discussion.

**Synthesis of Bi nanoparticles**

The Bi nanoparticles (NPs) were synthesized using the solvothermal route by following prior report.[^2^](#_ENREF_2) Briefly, the synthesis procedure was as follows: 1.5 mmol of bismuth (III) nitrate pentahydrate and 30 mL ethylene glycol were taken in a beaker. The solution was sonicated for 5 min to mix the precursors. Then, the solution mixture was transferred to a 50 mL Teflon vessel, and tightly packed with stainless steel autoclave. The autoclave was kept in a programmable oven at 210 ^o^C for 6 hours, followed by reaction mixture cooled to room temperature naturally. Subsequently, the obtained reaction mixture was washed with excess methanol using centrifugation at 1000 rpm for 8 min. This step was repeated twice. The obtained precipitate was placed in a vacuum oven at room temperature for overnight to dry the Bi NPs. The obtained Bi NPs was characterized using structural and microscopic techniques, used as catalyst in in-situ IR and ITC measurements.

Preparation of Bi NPs ink

Preparation of Bi NPs catalyst ink was as follows. Briefly, 20 mg of a fine grounded powder of the Bi NPs and 50 μL of Nafion solution were homogeneously dispersed in mixture of 3 : 1 ratio of ultra-pure water (0.75 mL), and isopropyl alcohol (0.25 mL). Then, the solution was taken to ultra-sonication until it formed homogeneous Bi NPs catalyst ink.

**Coating of Bi NPs on Glassy carbon (GC) and UiO-66 membrane formation on Bi NPs coated GC electrodes**

In order to prepare the Bi NPs catalyst on coated GC electrode, 10 μL of Bi NPs catalyst ink was drop-cast on GC electrodes. Subsequently, the electrodes were dried under ambient conditions for ~ 6 hours. All electrochemical experiments, the amount of catalyst loading was maintained at ∼ 0.2 mg. During the electrochemical experiments the exposed geometrical surface area was 0.196 cm^2^.

To form UiO-66 membrane on Bi NPs catalyst coated GC, the as-synthesized UiO-66 gel (50 µL) was diluted by adding 10 mL of ethanol. Thus, diluted homogeneous UiO-66 gel was used as a precursor to grow the UiO-66 membranes over Bi NPs catalyst coated GC. Precisely, the volume (10 µL) of diluted UiO-66 gel was drop-cost on Bi NPs catalyst coated GC. The time required to the formation of UiO-66 membrane on Bi NPs catalyst coated GC was ~3 to 4 hours at ambient conditions.

Electrochemical benzyl alcohol (BnOH) oxidation measurements

All electrochemical BnOH oxidation reactions were performed in 0.1 M H_2_SO_4_ aqueous electrolyte using a two-compartment cell with a conventional three-electrode system. Bi-foil and UiO-66 coated Bi foil (Bi-UiO-66) electrodes were used as the working electrode in different experiments. Pt flag and Ag/AgCl electrodes were used as counter and reference electrodes. An ion-conducting Nafion membrane separated the working compartment and the counter compartment. The anodic compartment, i.e., the compartment containing the working electrode, was filled with 50 mL of 0.1 M H_2_SO_4_ solution for an electrochemical BnOH oxidation reaction. All the electrochemical experiments were performed under an inert atmosphere. Separate measurements were done to determine the Faradaic efficiency (FE) of benzaldehyde and benzoic acid products.

The electrochemical BnOH oxidation reaction was performed for the quantification of benzaldehyde and benzoic acid. During the electrolysis, the electrolyte solution in the working compartment was constantly stirred (600 rpm). A constant charge of 1.5 C was passed through the working electrode at different potentials. After the electrolysis, the electrolyte solution from the working compartment was collected and stored in sealed containers in a refrigerator (4 °C - 8 °C). Then, 400 μL of the electrolyte solution was taken from the stock electrolyte solution for the water separation NMR analysis.

Quantification of benzoic acid from 1H NMR

We have quantified the benzoic acid using 1H NMR signal from the ortho proton of benzoic acid. For the same proton, first, we produced the calibration curve (Figure S13) using a known concentration of benzoic acid, where the experimental data points linearly fit with the increasing concentration of benzoic acid. Using the calibration data, we have quantified the unknown concentration of benzoic acid after the electrolysis. Faradic efficiencies were determined using the below formulae.

Faradic efficiency (FE) = nF × (m/Q)

Where, n = number of electrons involved in the reaction,

F= Faraday constant,

m = moles of product formed,

Q = applied charge.

Thus, the modified equation: FE-benzaldehyde = (2 × 96485 C × (n-benzaldehyde / 1.5 C)), Where, n-benzaldehyde: moles of benzaldehyde formed.

likewise, the modified equation: FE-benzoic acid = (4 × 96485 C × (n-benzoic acid / 1.5 C)), where n-benzoic acid: moles of n-benzoic acid formed.

Experimental details for two electrode measurements

We integrated two electrode electrolyzers to determine FE for H_2_ evolution by separating an ion exchange membrane. 50 mL of 0.3 M BnOH electrolyte solution was taken in the anode compartment, and 50 mL electrolyte solution was taken in the cathode compartment. The compartments were purged with Argon for 40 min, and then the cell was sealed without a leakage. The electrolyte solution in the working compartment was constantly stirred (600 rpm) during the electrolysis. A constant potential (0.65V) was applied to the working electrode for 3 hours. The gaseous product (H_2_) was collected at different time intervals from the sealed head-space with a Hamilton gas syringe and analyzed by gas chromatographic technique (Agilent). And the Faradic efficiency of H_2_ was calculated using the below formulae.

Faradic efficiency (FE) = nF × (m/Q).

Where, n = number of electrons involved in the reaction,

F= Faraday constant,

m = moles of product formed

Q = Applied charge

Thus, the modified equation: FE-H_2_ = (2× 96485 C × (n-H_2_ / applied charge (C))),

Where, n-H_2_: moles of H_2_ formed.

ATR-IRRAS (attenuated total reflectance surface-enhanced infrared absorption spectroscopy in Otto configuration) experimental details

ATR measurements were carried out using the Thermo Nicolet iS50 instrument equipped with liquid nitrogen cooled MCT-A detector with a CaF_2_ window (4000 - 800 cm^-1^). A custom-made spectro-electrochemical cell supplied by the Beijing Scistar technology (Co. Ltd.) was mounded on PIKE instrument VeeMAXIII ATR optical accessory. All the ATR experiments were performed using CaF_2_. IR data were collected and analyzed using the software OMNIC SPECTRA, supplied by Thermo Fischer Scientific. The electrochemical cell was composed of a typical three electrode design. The working electrode was aligned tightly above the CaF_2_ window with the help of an O-ring. The reference and counter electrodes were placed on either side of the working electrode and were not exposed to the incident IR beam. Ag/AgCl electrode and a Pt foil were used as a reference, and counter electrode, respectively, for the measurements. Bi NPs or Bi NPs-UiO-66 coating on glassy carbon was used as a working electrode. A thin solution layer was present between the working electrode (Bi NPs or Bi NPs-UiO-66) and the CaF_2_ window, and it was exposed to the incident IR light. The working electrode surface and the thin layer solution between the IR window and the working electrode were studied by the ATR-IRRAS experiments. The electrolyte solution was continuously flowed within the cell by solution inlet and outlet from the stock reservoir using a pump.

Isothermal titration calorimetry (ITC)

ITC measurements were performed on a Nano ITC calorimeter by TA Instruments, having 20 injection cycle method, with sample cell size (1 mL), sample volume (1 mL) and injection syringe volume (50 µL) for each set of experiment. In a typical ITC experiment, the reference cell is filled with deionized water. The sample cell was filled with a catalyst suspension, and the syringe is loaded with a benzaldehyde solution. Bi, Bi-UiO-66 catalyst suspension and benzaldehyde solutions were prepared in DDW water with a concentration of 0.05 mM and 0.5 mM, respectively. Samples were degassed for 20 min, before collecting the thermograms. All thermograms were collected at a fixed stirring rate of 250 rpm. Incremental titration injection with 20 aliquots, the first of 1.25 µL and the next 19 aliquots of 2.5 µL each, with injection intervals of 350 sec. All experiments were performed at 298 K. The heats of injection for blank titrations (benzaldehyde into water) were subtracted from the heats of injection into Bi and Bi-UiO-66 suspensions. And, data analysis was performed using TA NanoAnalyze data Analysis software.

Calculation of density of Zr_6_ nodes in Bi-UiO-66

For the ICP-OES analysis, a known amount of UiO-66 gel was digested in 5 mL of concentrated nitric acid (HNO_3_) at 120 ℃ for 12 hours. 1 mL of the acid solution was diluted to 10 mL by addition of water and analyzed using ICP-OES. The concentration of Zr_6_ nodes was determined from the experimentally obtained Zr ions in the 10 mL solution.

UiO-66 molecular formula: Zr_6_O_4_(OH)_4_(OOC-C_6_H_4_-COO)_6_; molar mass: 1664.06 g/mole.

The density of Zr in UiO-66 was obtained from ICP-OES = 11.027 mg/L.

3.11 mg of UiO-66 contains 0.590 mg of Zr.

3.11 mg of UiO-66 contains 10.6×10^-7^ moles of Zr_6_.

3 mg of UiO-66 contains 10.225 ×10^-7^ moles of Zr_6_.

Determined the density of missing linker defect in UiO-66 was using ICP-OES and ^1^H-NMR analysis.

Amount of BDC found in 3 mg of UiO-66 = 41.8 × 10^-7^ mole.

Moles Zr_6_ obtained from ICP-OES analysis for UiO-66, BDC/Zr_6_ = 4.08.

Thus, the number of BDC per node = 8.16 ≈ 8 for UiO-66 gel.

So, the number of defect sites per node = (12 – 8) = 4 for UiO-66 gel.

Note: The density of Zr_6_ nodes for Bi-UiO-66 should be identical as that of UiO-66 gel.

## Physical characterization methods

The structural information of UiO-66 membranes was analyzed from powder x-ray diffraction pattern (PXRD) using PAN analytical's Empyream multi-purpose diffractometer instrument and Cu-Kα (1.5405 Å) radiation.

The scanning electron microscope (SEM) images of the UiO-66 membrane were captured at different magnifications using the Verios XHR 460L SEM instrument. All the UiO-66 membranes on the Bi foil were gold coated (9 nm) before being subjected to the SEM measurements.

The SEM-FIB (SEM-focused ion beam) images were taken using a Thermo Scientific Dual-Beam system. The ion beam and the electron intersect at a 52° angle near the sample surface. The UiO-66 membranes on the Bi foil were gold coated in a thick layer of 30 nm and then analyzed using SEM-FIB. During the analysis, the area under investigation was coated first with 150 nm of Pt and, on top of that, 1500 nm of carbon by ion beam assisted chemical vapor deposition. Next, a small area was cut by the sputtering of the focused ion beam to visualize the cross-sectional thickness of the membrane.

Hydrogen Nuclear Magnetic Resonance (H-NMR) experiments were performed using the Bruker DPX-500 instrument. A calibration curve was prepared using a different known concentration of 1, 4 benzene dicarboxylic acid (H_2_BDC) to determine the amount of BDC in UiO-66 gel. The standard H_2_BDC samples were prepared by digesting in 1 M NaOH/D_2_O. A known amount of trimesic acid was used as the internal standard for each NMR experiment. The amount of BDC was determined by digesting a known amount of UiO-66 in 1 M NaOH/D_2_O solution.

The amount of Zr in UiO-66 was predicted from the Inductively coupled plasma-optical emission spectroscopy (ICP-OES) using Spectro ARCOS ICP-OES, FHX22 multi-View plasma (SOP, EOP) instrument. The sample preparation was as follows. First, 3.3 mg of UiO-66 was digested in 5 ml of concentrated nitric acid (HNO_3_) at 120 ℃ for 12 hours. 1 mL of this digested solution was diluted to 10 mL water for ICP-OES experiments.

The X-ray photoelectron spectroscopy (XPS) data of UiO-66 membranes on Bi foil were acquired using ESCALAB 250 apparatus X-ray photoelectron spectrometer, with Al-Kα X-ray source and monochromator. The survey spectra were obtained with a pass energy (PE) of 150 eV, and a high energy resolution was achieved with a PE of 20 eV. XPS spectra of samples were corrected with respect to the carbon 1s binding energy (284.6 eV) as a reference.

All the electrochemical experiments were conducted using a BioLogic VSP-128 electrochemical workstation.

ITC measurements were performed on a Nano ITC calorimeter by TA Instruments.

Gaseous products were analyzed using Gas chromatography (GC). Pressure Lok Precession Analytical Syringe was used to carry out measurements. In each injection, 500 μL of the head-space gas mixture was injected manually after the electrolysis. The data was analyzed and quantified using GC, considering head space as an accumulated gas volume. The GC measurements were carried out on PerkinElmer Clarus 590 GC equipped with a wide-range flame ionization detector (FID), methanizer, and thermal conductivity detector (TCD).

RESULTS AND DISCUSSION

**Figure S1.** (a), (b), and (c) presents the top-view SEM images of Bi-UiO-66 at different magnifications.

Figure S2. (a) XPS surface survey spectrum of (a) Bi-UiO-66 and (b) after etching UiO-66 on Bi with ion beam.

Figure S3. XPS spectra of Bi 4f for Bi foil and Bi-UiO-66.


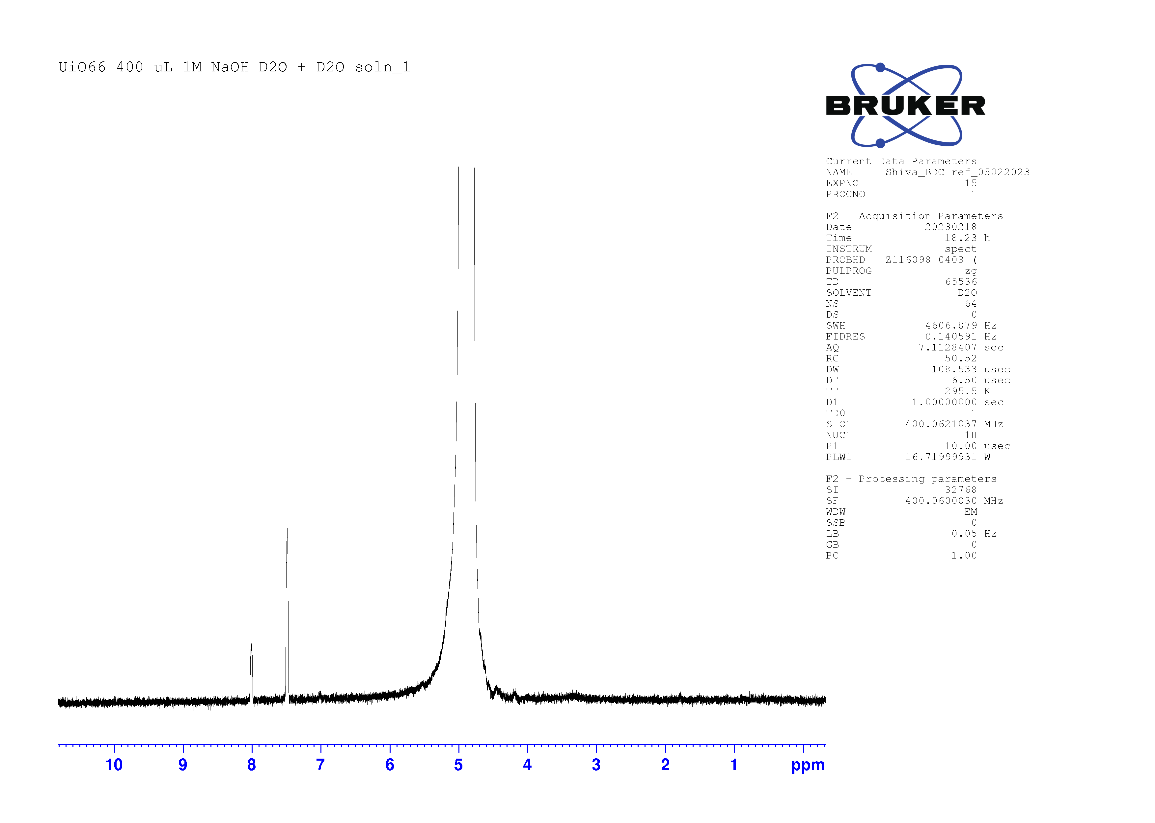
Figure S4. 1H-NMR spectrum of BDC after digesting known amount of UiO-66 in 1 M NaOH (D_2_O) solution. Trimesic acid was used as internal standard.

Figure S5. 1H-NMR calibration plot was prepared using a known concentration of benzene dicarboxylate (BDC) for the calculation of the amount of BDC in UiO-66.

Figure S6. Cyclic voltammogram data for Bi foil in 0.1 M H_2_SO_4_ aqueous electrolyte solution at a scan rate of 50 mV/s. Ag/AgCl and Pt flag electrodes were used as a reference and the counter electrode, respectively.

Figure S7. Chronoamperometric experiments were performed on Bi foil at different applied potentials (a) from 0.275 to 0.5 V vs. RHE, and (b) corresponding Q versus time plots.

Figure S8. Chronoamperometric experiments were performed on Bi-UiO-66 different applied potentials (a) from 0.275 to 0.5 V vs. RHE, (b) corresponding Q versus time plots.

Figure S9. (a) and (b) the total Faradic efficiencies of (benzaldehyde + benzoic acid) for Bi-foil, and Bi-UiO-66, respectively. Electrolysis was performed in the potential range 0.45 V to 0.5 V (vs. RHE) 0.3 M BnOH in 0.1 M H_2_SO_4_ aqueous electrolyte solution.

Figure S10. 1H-NMR calibration plot was prepared to determine the Faradic Efficiencies (FE) for benzaldehyde production for Bi and Bi-UiO-66 catalysts. Note: calibration plot was obtained from a different known concentration of benzaldehyde samples in the same electrolyte solution using NMR.

Figure S11. 1H-NMR calibration plot was prepared to determine the Faradic Efficiencies (FE) for benzoic acid production for Bi and Bi-UiO-66 catalysts. Note: calibration plot was obtained from a different known concentration of benzoic acid samples in the same electrolyte condition using NMR.


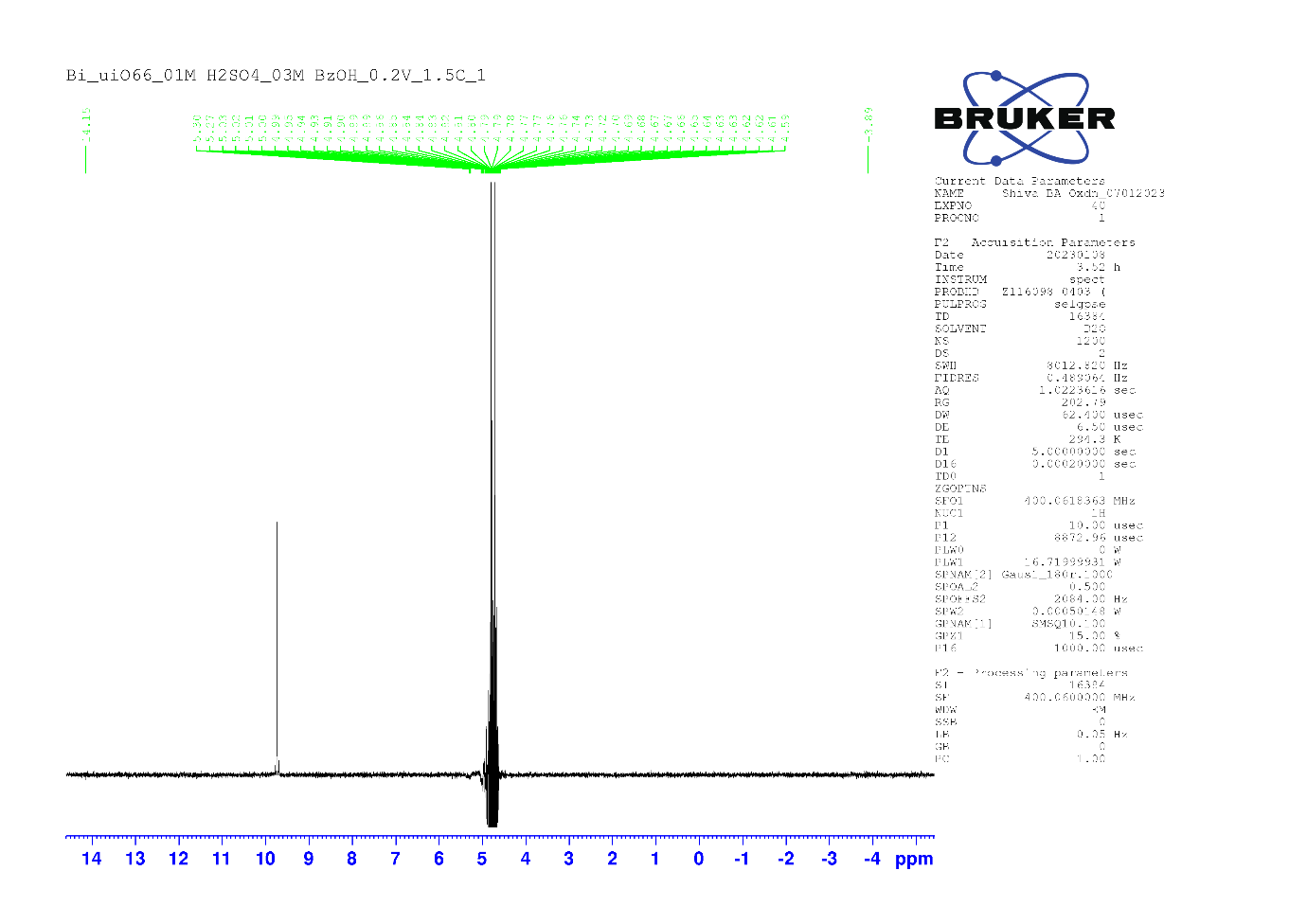


Figure S12. A representative 1H-NMR spectrum was obtained for benzaldehyde quantification in the case of Bi-UiO-66 used as the catalyst for the benzyl alcohol oxidation reaction.


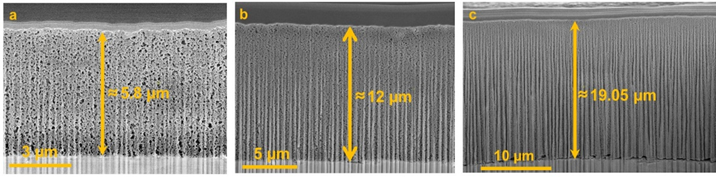


Figure S13. SEM-FIB cross section image of different thickness of UiO-66 membrane on Bi electrocatalyst.


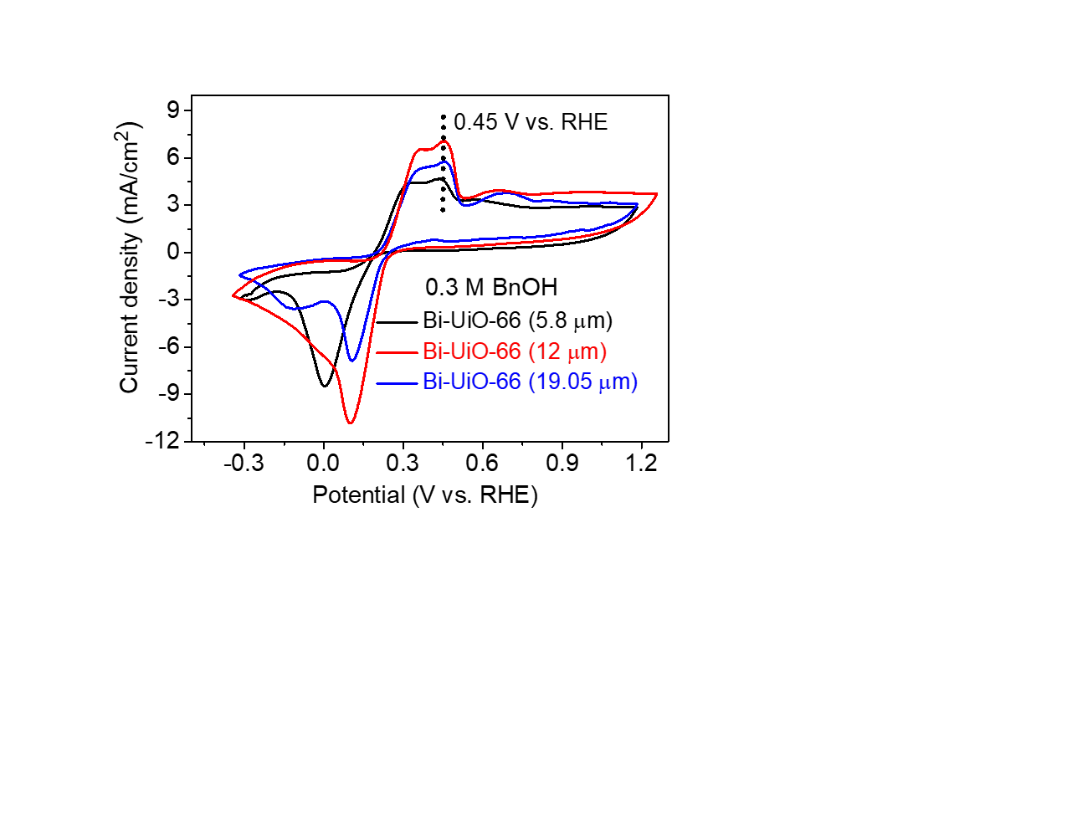


Figure S14. CV data of different thickness of UiO-66 membrane on Bi electrocatalyst.


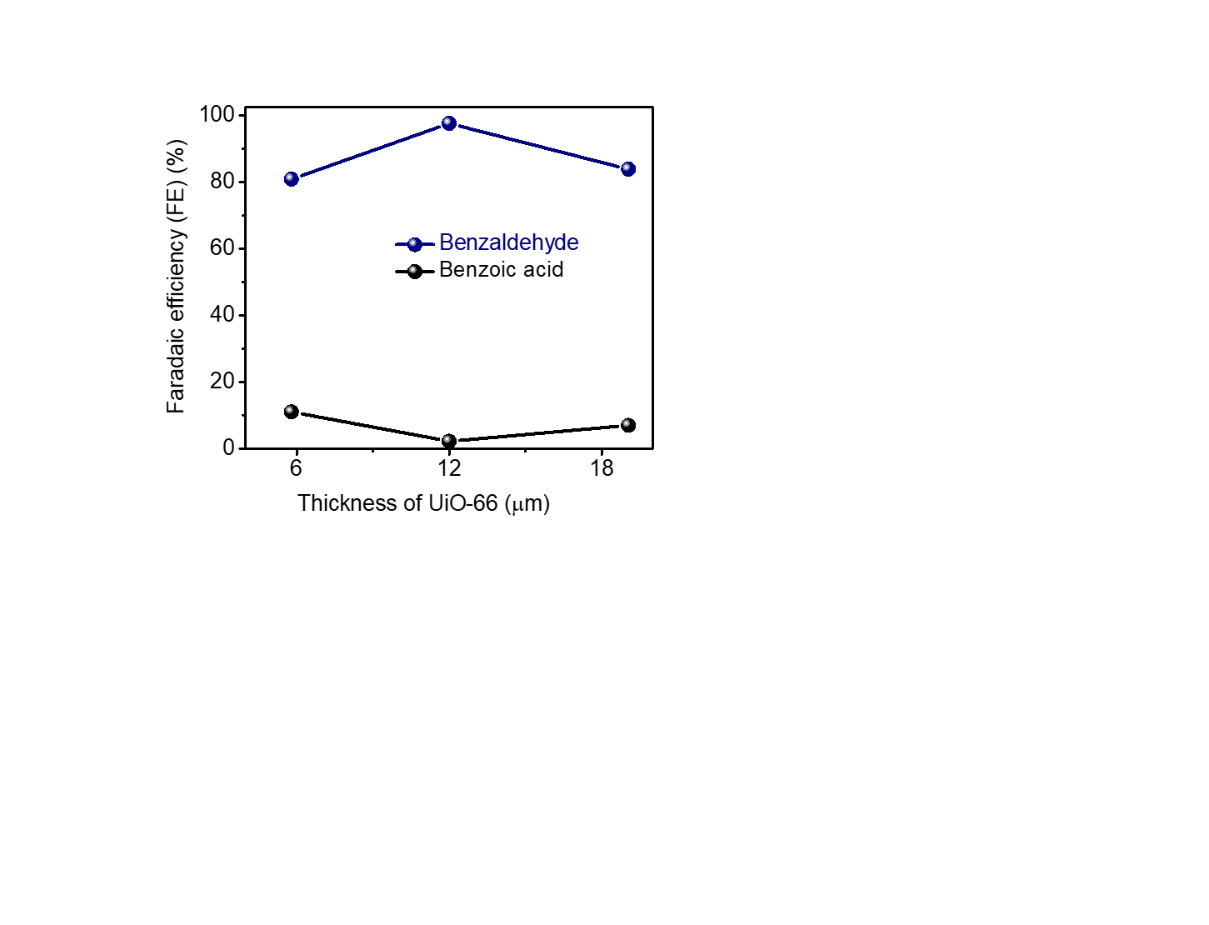
Figure S15. Comparison of UiO-66 thickness on Bi foil versus faradaic efficiency for BnCHO and BnCOOH at the applied potential 0.45 V vs. RHE.


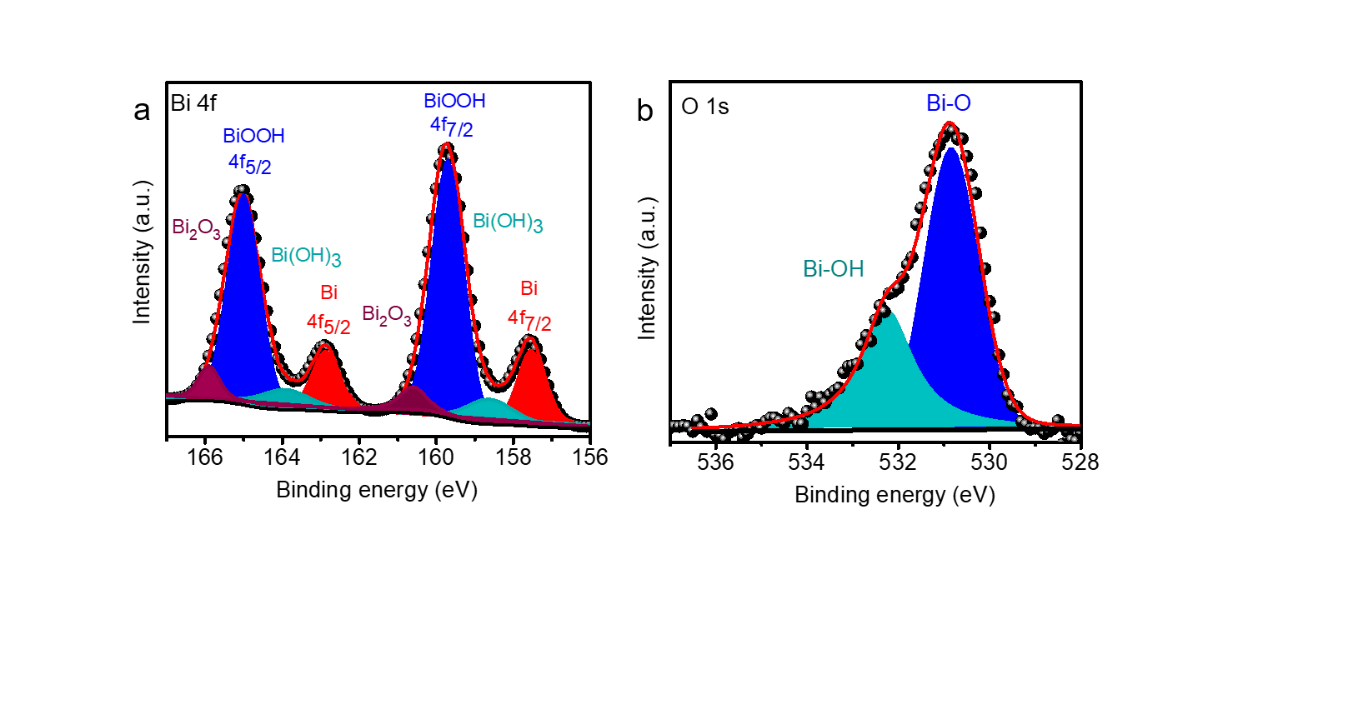


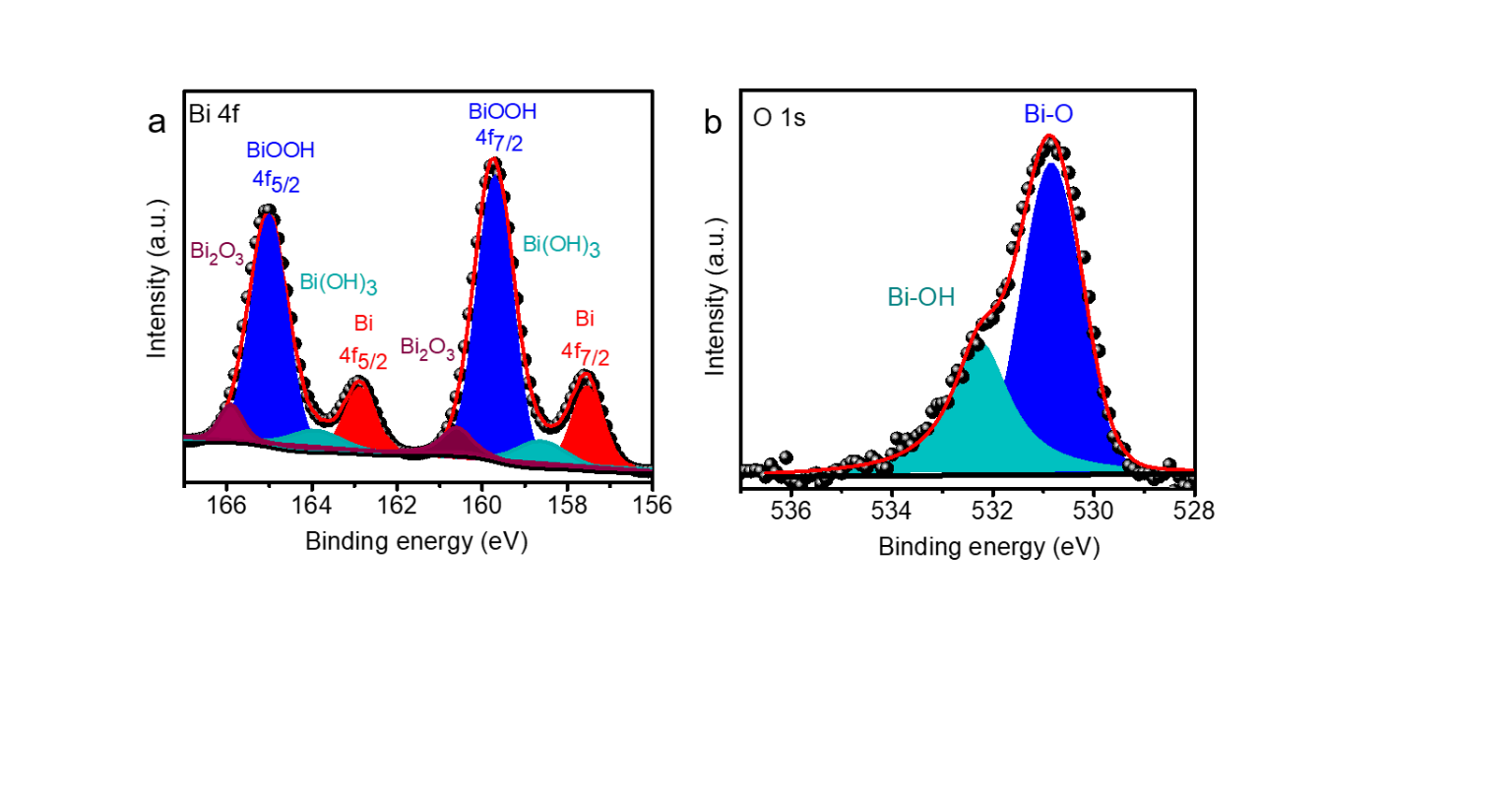
Figure S16. Surface characterization of Bi-UiO-66 electrode after passing 1.5 C charge at 0.45V vs RHE. High resolution XPS spectra of (a) Bi 4f, and (b) O 1s.

Figure S17. Chronoamperometric measurement was performed on Bi-UiO-66 at the 0.45 V vs. RHE.

Figure S18. Comparison of PXRD pattern of Bi-UiO-66 before and after bulk electrolysis for 5 hours along with reference pattern of UiO-66.

Figure S19. SEM (top view) images of Bi-UiO-66 after bulk electrolysis for 5 hours.


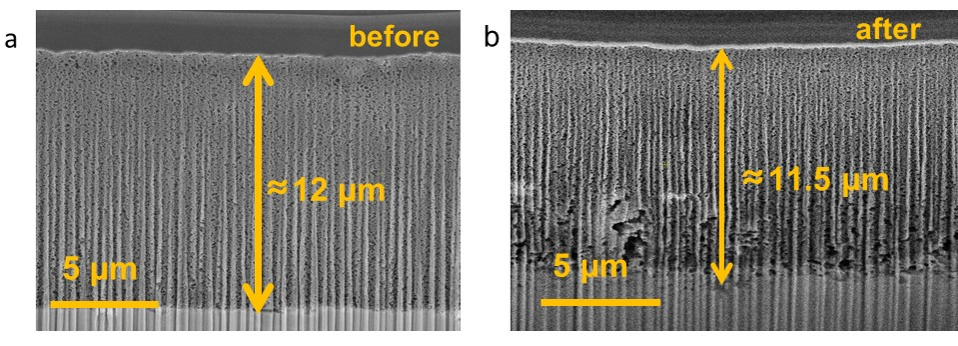


Figure S20. SEM-FIB of Bi-UiO-66 after bulk electrolysis for 5 hours.


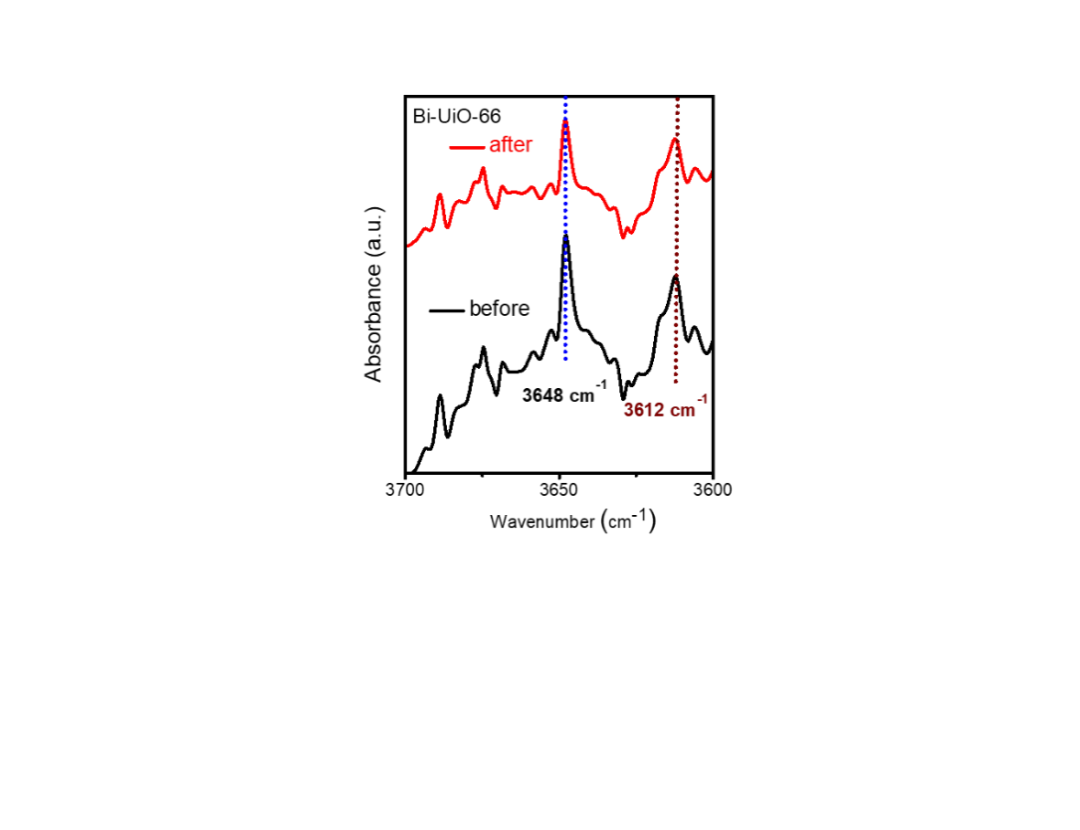


Figure S21. DRIFTS spectra of Bi-UiO-66 after bulk electrolysis for 5 hours.


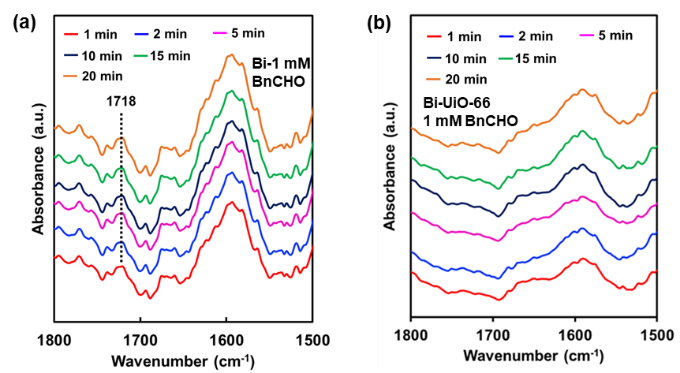


**Figure S22.** High frequency region of the FTIR data of (a) Bi and (b) Bi-UiO-66 in presence of 1 mM BnCHO electrolyte solution.

Figure S23. Chronoamperometric measurement was performed on Bi-UiO-66 in a two-electrode design at applied potential of 0.65 V.


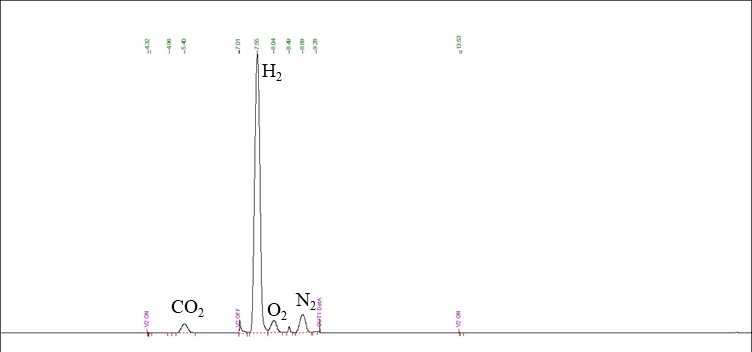


Figure S24. A representative GC spectrum was obtained for H_2_ quantification where Bi-UiO-66 was used as a catalyst for the benzyl alcohol oxidation reaction in the two-electrode electrolysis.

**Figure S25.** CA data of Bi-UiO-66 catalyst at 0.45 V vs. RHE in a 0.1 M H_2_SO_4_ with 0.3 M BnOH electrolyte solution.

In order to understand whether the decrease of current density with the function of time in a chronoamperometric (CA) experiment (Figure S24) is because of catalyst deactivation or reactant consumption during the electrolysis, we conducted another set of CA experiment on Bi-UiO-66 catalyst at 0.45 V vs. RHE under the same experimental condition. As CA progressed, the current density was decreased partially in 1 hour. Then, we injected fresh BnOH solution into the working compartment of the electrochemical cell, and the current density was restored (Figure S24, 1^st^ addition). After decreasing the current density 2^nd^ time at 2.5 hours, again, we injected fresh BnOH solution into the working compartment (Figure S24, 2^nd^ addition), which resulted in increase in the current density back to almost the same value at the initial stage of the reaction. Therefore, it clearly suggests that the decrease in current density with time in long-term electrolysis is mainly because of the consumption of reactant but not the deactivation of the catalyst. However, the partial deactivation of the catalyst cannot be ruled out in long-term electrolysis.

In the present report, the top surface Bimetal foil is used as an electrocatalyst, and all other faces are masked with insulating tape. Surface active sites are calculated on Bi using the area under the curve by integrating peak at 0.375 V RHE in cyclic voltammetry data (Figure 3a), recorded in a bare electrolyte solution. The surface coverage of -OOH species on Bi electrocatalyst is calculated to 1.09 x 10^-8^ moles cm^-2^. The turnover number (TON) calculated for the product benzaldehyde is 3009, and the corresponding turnover frequency (TOF) is 0.27 s^-1^. Hence, the catalyst doesn’t degrade very quickly to be enable to remain active until the presence of BnOH in the system, signifying that the electrooxidation reaction is a catalytic reaction not a stoichiometric one.

**Table S1.** Thermodynamic parameters of adsorption of benzaldehyde on Bi and Bi-UiO-66.

| Catalyst | ∆H (kJ/mol) | K_a_ (M^-1^) | T ∆S (kJ/mol) | ∆G (kJ/mol) |
| --- | --- | --- | --- | --- |
| Bi | -53.4 | 1.379 x 10^6^ | 18.7 | -34.7 |
| Bi-UiO-66 | -5.89 | 0.598x 10^6^ | -26.5 | -32.6 |

**REFERENCES**

(1) Bueken, B.; Van Velthoven, N.; Willhammar, T.; Stassin, T.; Stassen, I.; Keen, D. A.; Baron, G. V.; Denayer, J. F. M.; Ameloot, R.; Bals, S.; De Vos, D.; Bennett, T. D. Gel-Based Morphological Design of Zirconium Metal-Organic Frameworks. *Chem. Sci.* **2017,** *8* (5), 3939-3948, DOI: 10.1039/C6SC05602D.

(2) Zhang, M.; Feng, C.; Zhang, W.; Luan, X.; Jiang, J.; Li, L. Synthesis of Bismuth Nanoparticles by a Simple One-Step Solvothermal Reduction Route. *Appl. Mech. Mater.* **2013,** *423-426*, 155-158, DOI: 10.4028/[www.scientific.net/AMM.423-426.155](http://www.scientific.net/AMM.423-426.155).

**Author Contributions**

Idan Hod supervised the project. G. Shiva Shanker carried out the project. G. Shiva Shanker conducted all the experiments and analyzed data. Arnab Ghatak helped with insitu IR analysis. Ran Shimoni helped with NMR and gas-chromatography experiments. Itamar Liberman carried out the ICP-OES and SEM measurements. Shahar Binyamin performed the XRD measurements. Idan Hod and G. Shiva Shanker wrote the paper.
